# Radiomics Nomogram for Identifying Sub-1 cm Benign and Malignant Thyroid Lesions

**DOI:** 10.3389/fonc.2021.580886

**Published:** 2021-06-07

**Authors:** Xinxin Wu, Jingjing Li, Yakui Mou, Yao Yao, Jingjing Cui, Ning Mao, Xicheng Song

**Affiliations:** ^1^ Department of Otorhinolaryngology-Head and Neck Surgery, Yantai Yuhuangding Hospital, Qingdao University, Yantai, China; ^2^ School of Clinical Medicine, Binzhou Medical University, Yantai, China; ^3^ Collaboration Department, Huiying Medical Technology Co., Ltd, Beijing, China; ^4^ Department of Radiology, Yantai Yuhuangding Hospital, Qingdao University, Yantai, China

**Keywords:** nomogram, radiomics, computed tomography, thyroid imaging reporting and data system, thyroid lesions

## Abstract

**Purpose:**

To develop and validate a radiomics nomogram for identifying sub-1 cm benign and malignant thyroid lesions.

**Method:**

A total of 171 eligible patients with sub-1 cm thyroid lesions (56 benign and 115 malignant) who were treated in Yantai Yuhuangding Hospital between January and September 2019 were retrospectively collected and randomly divided into training (n = 136) and validation sets (n = 35). The radiomics features were extracted from unenhanced and arterial contrast-enhanced computed tomography images of each patient. In the training set, one-way analysis of variance and least absolute shrinkage and selection operator (LASSO) logistic regression were used to select the features related to benign and malignant lesions, and the LASSO algorithm was used to construct the radiomics signature. Combined with clinical independent predictive factors, a radiomics nomogram was constructed with a multivariate logistic regression model. The performance of the radiomics nomogram was evaluated by using the receiver operating characteristic (ROC) and calibration curves in the training and validation sets. The clinical usefulness was evaluated by using decision curve analysis (DCA).

**Results:**

The radiomics signature consisting of 13 selected features achieved favorable prediction efficiency. The radiomics nomogram, which incorporated radiomics signature and clinical independent predictive factors including age and Thyroid Imaging Reporting and Data System category, showed good calibration and discrimination in the training (area under the ROC [AUC]: 0.853; 95% confidence interval [CI]: 0.797, 0.899) and validation sets (AUC: 0.851; 95% CI: 0.735, 0.931). DCA demonstrated that the nomogram was clinically useful.

**Conclusion:**

As a noninvasive preoperative prediction tool, the radiomics nomogram incorporating radiomics signature and clinical predictive factors shows favorable predictive efficiency for identifying sub-1 cm benign and malignant thyroid lesions.

## Introduction

According to ultrasound (US) screening and autopsy studies, thyroid lesions, which mainly include benign lesions and thyroid cancer, are common diseases with a prevalence of 30%–67% in the general population (1, 2). Papillary thyroid microcarcinoma (PTMC) is a subtype of papillary thyroid carcinoma (PTC), which is defined by the WHO as having a maximum diameter of 1.0 cm or less (3, 4). In recent decades, the incidence of thyroid cancer has rapidly increased throughout the world (5–7), with PTMC accounting for half of new cases (3, 8, 9). Although PTMCs usually have an indolent course, 24%–63% of patients may develop cervical regional lymph node metastasis at presentation (10, 11). To avoid overtreatment of thyroid lesions, benign lesions should be accurately distinguished from malignant ones before performing a biopsy or surgical resection (8, 9, 12).

At present, the main methods used to diagnose thyroid lesions are US and US-guided fine-needle aspiration biopsy (US-FNAB) (13, 14). However, US examinations show a diagnostic sensitivity of only 27%–63% for detecting lesion malignancy and are highly dependent on radiologists’ experience (15). Previous studies have shown that different radiologists can make different diagnoses after reviewing the US images of the same thyroid nodule (16). US-FNAB has a sensitivity of 54%–90% and a specificity of 60%–98% in diagnosing PTMC, and it has a sensitivity of approximately 30% in detecting non-diagnostic and indeterminate lesions (17–19). The American Thyroid Association guidelines do not recommend biopsy for sub-1 cm lesions that are highly suspicious for PTC on US. No non-invasive method can effectively and reliably diagnose PTMC. Thus, the methods for diagnosing sub-1 cm thyroid lesions should be improved, and the need for biopsy and diagnostic surgery should be reduced.

Computed tomography (CT), a common imaging examination method, is of great auxiliary value in preoperatively evaluating and determining the extent, localization, and lymph node status of the tumor (20). However, most diagnostic information from CT is based on visual inspection by a radiologist, who may miss critical diagnostic information. Thus, conventional CT is not effective in diagnosing thyroid lesions, especially sub-1 cm ones (21). In recent years, radiomics, which is the quantitative analysis of a large amount of data in medical images by means of computer technology, has received increasing attention due to its improved diagnosis and prediction accuracy (22–27). When combined with other relevant clinicopathological variables, radiomics-derived data can produce a more accurate and robust evidence-based decision system (28). Although the radiomics features of CT images can be used to help radiologists identify benign and malignant thyroid lesions (29), to the best of our knowledge, no radiomics-based study has predicted sub-1 cm benign and malignant thyroid lesions.

Therefore, the present study aimed to develop and validate a radiomics nomogram that incorporates radiomics features and clinical risk factors for identifying sub-1 cm benign and malignant thyroid lesions.

## Materials and Methods

### Patients

**Figure 1:**
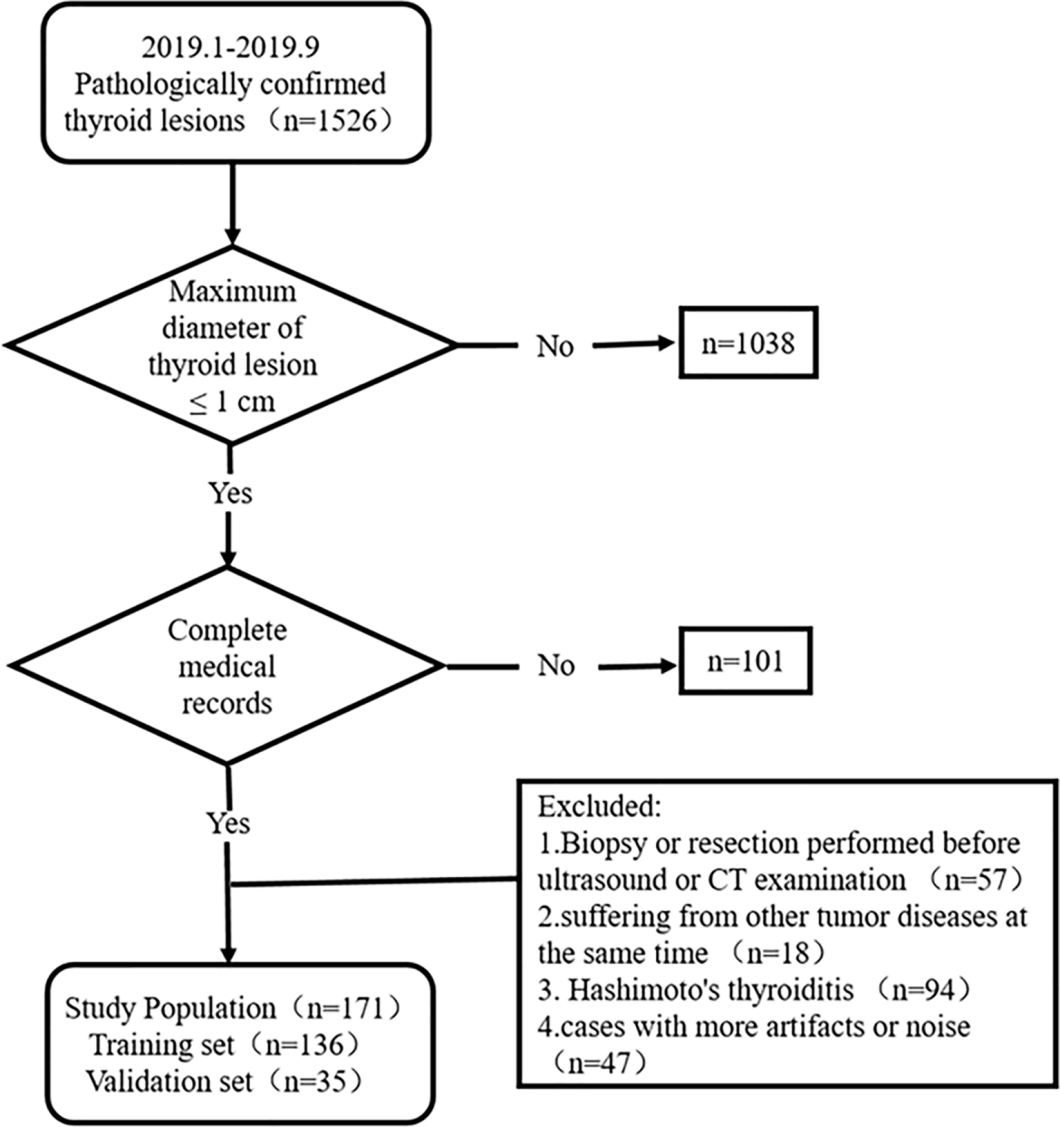
Recruitment pathways for patients.

This study was approved by the ethics committee of the Yantai Yuhuangding Hospital. The informed consent requirement was waived. Patients with thyroid lesions who were treated at Yantai Yuhuangding Hospital from January to September 2019 were consecutively collected according to the inclusion and exclusion criteria. The inclusion criteria were as follows: (1) the pathology of surgical specimens was certain; (2) the maximum diameter of the thyroid lesion was ≤1 cm; and (3) clinical, US, and CT data were complete. The exclusion criteria were as follows: (1) biopsy or resection had been performed before the US and CT examination, (2) patients suffering from other tumor diseases, (3) patients with Hashimoto’s thyroiditis, and (4) cases with artifacts or noise affecting image quality. **Figure 1** shows the recruitment pathway of patients. A total of 171 fully eligible patients with sub-1 cm thyroid lesions met the criteria (mean age, 46.47 ± 11.03 years; range, 21 to 71 years) were included. The patients were divided into two sets at a ratio of 8:2 using computer-generated random numbers: training set (n = 136; mean age, 46.21 ± 11.18 years; range, 21 to 71 years) and independent validation set (n = 35; mean age, 47.46 ± 10.54 years; range, 25 to 63 years).

The clinical data of each patient were obtained by reviewing the medical records, including age, gender, Thyroid Imaging Reporting and Data System (TI-RADS) category, CT characteristics (maximum diameter, calcification, and location of nodule), free triiodothyronine (FT3), free thyroxine (FT4), and thyroid-stimulating hormone (TSH). Two senior radiologists reviewed all images and reassessed each lesion according to the 2017 American College of Radiology TI-RADS scoring criteria. The CT characteristics were re-examined and recorded by two radiologists with 10 years (Dr. A) and 8 years (Dr. B) of experience in the diagnosis of thyroid lesions. Any disagreements were resolved through negotiation to ensure accuracy and repeatability.

### CT Image Acquisition

All patients underwent contrast-enhanced thyroid CT with a 64-slice spiral CT scanner (Siemens, Germany) or 256-slice spiral CT scanner (Philips, Netherlands). The exposure parameters for the CT scan were as follows: 120 kV, 300 effective mAs, scanning slice thickness 1.25 mm, pitch of 0.97, and matrix of 512 × 512. The scan range was from the skull base to the subclavian region. After unenhanced CT scanning, a contrast-enhanced CT scan was performed. Approximately 80–100 ml of nonionic contrast material (iopamidol, 320 mg/ml) was injected into the cubital vein at a rate of 3.5 ml/s, and then saline (30 ml) was injected at the same rate. Arterial-phase images were obtained at 30 s. All images were derived from the Picture Archiving and Communication System with the data format of Digital Imaging and Communications in Medicine. The images were imported into Radcloud (Huiying Medical Technology Co., Ltd.) and preprocessed. This process consisted of three steps, namely, standardization of the gray value of the region of interest (ROI), discretization of the gray level, and image resampling (30–32).

### ROI Segmentation, Radiomics Feature Extraction, and Radiomics Signature Construction

**Figure 2:**
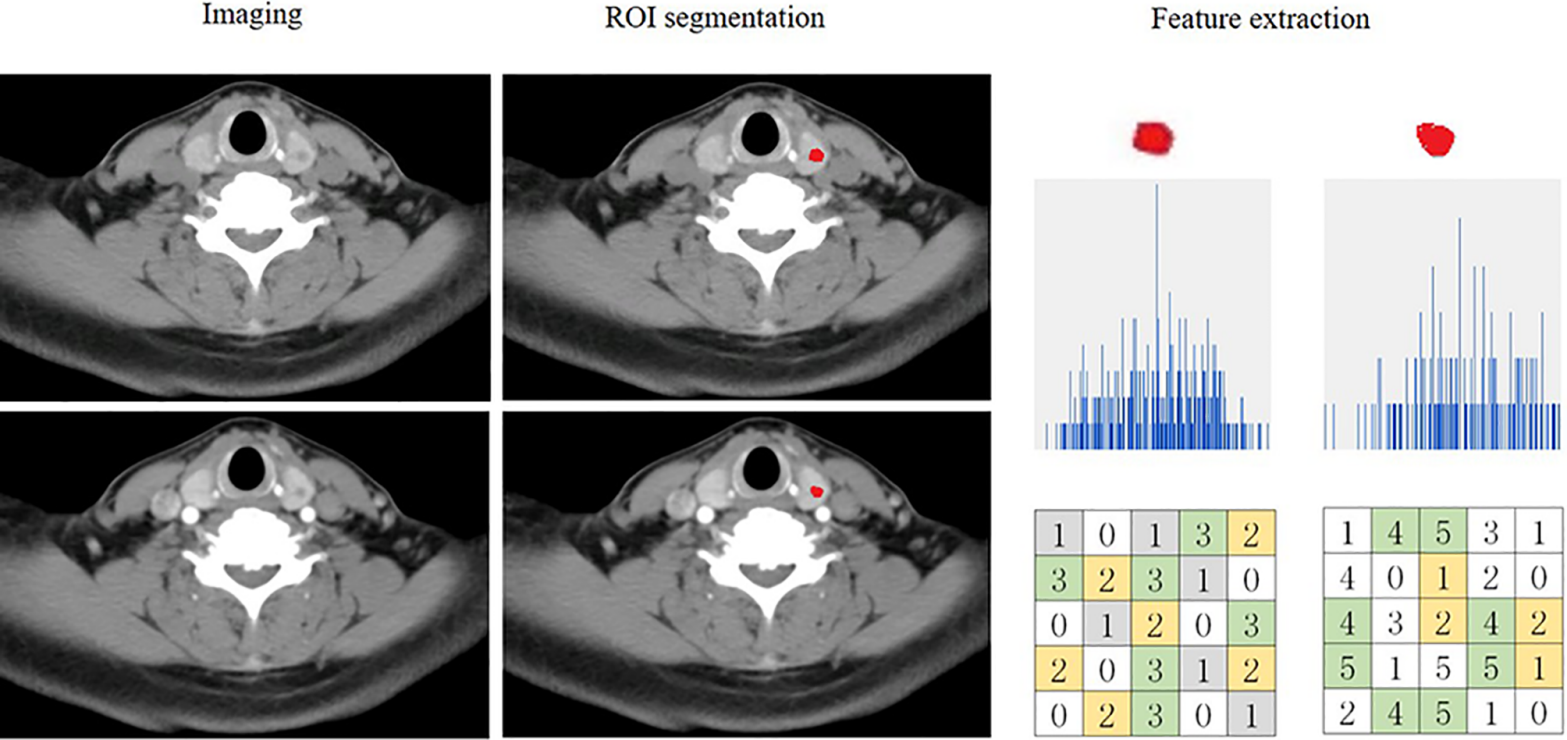
Flowchart showing the process of radiomics. An example of imaging segmentation and features extraction for patients with malignant nodule. ROI segmentation is performed on unenhanced and arterial contrast-enhanced computed tomography images. Features are extracted from the ROI, including tumor shape, intensity and texture. ROI, region of interest.

**Figure 3:**
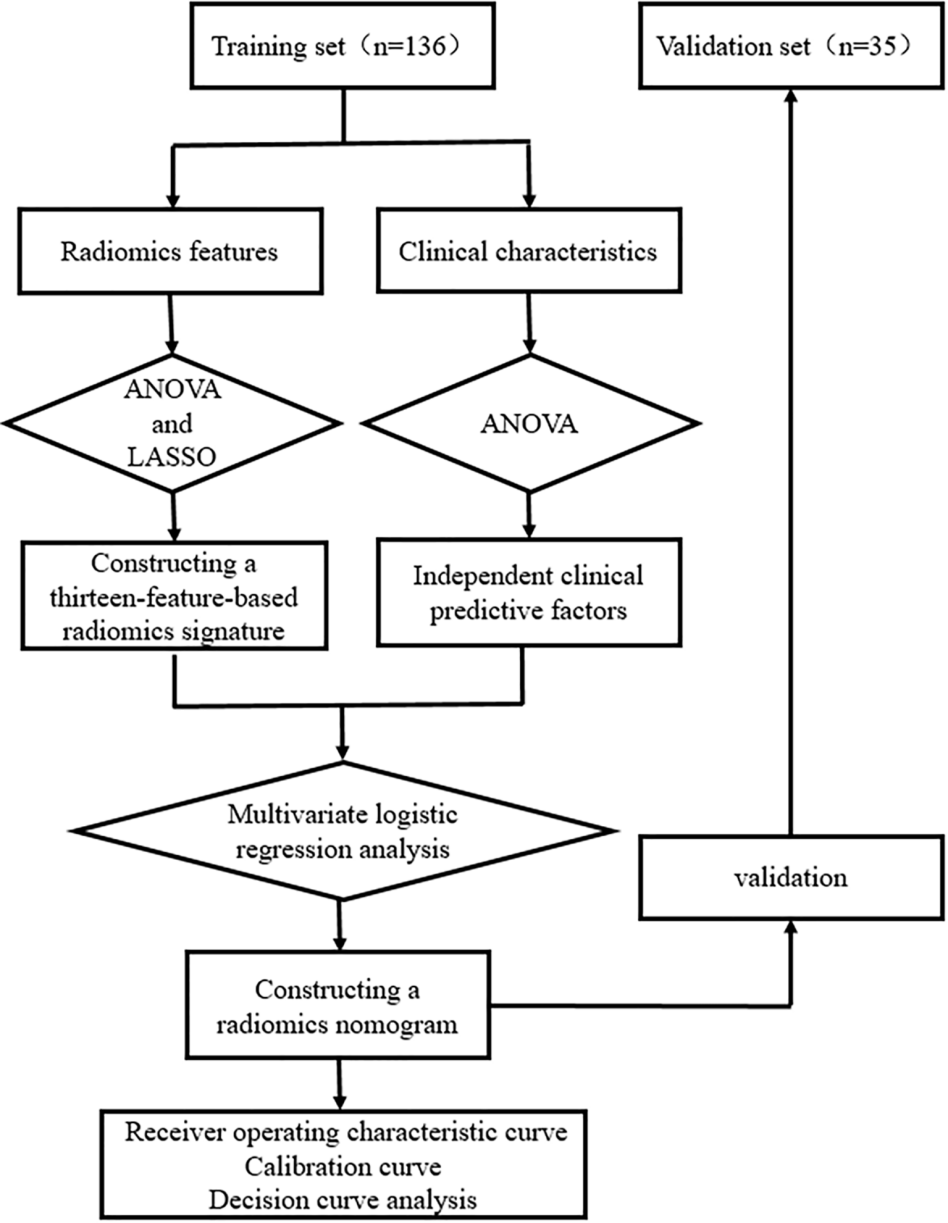
Study method flowchart.

**Figures 2** and **3** present the radiomics workflow and study flowchart. The tumor ROI was manually segmented on the unenhanced and arterial contrast-enhanced CT images of the largest cross-sectional section. The manual segmentations were performed by Dr. A and Dr. B who were blinded to the pathologic results. Radiomics features (shape, firstorder, texture features) were extracted automatically from the ROIs of each image. ROI segmentation and radiomics feature extraction were performed using Radcloud (Huiying Medical Technology Co., Ltd.).

Intra- and inter-class correlation coefficients (ICCs) were used to evaluate the intra- and inter-observer reproducibility of radiomics feature extraction. First, Dr. A and Dr. B randomly analyzed the images of 30 patients to evaluate the inter-class reproducibility. Two weeks later, Dr. A repeated the same procedure. An ICC greater than 0.8 indicates good agreement of the feature extraction. The remaining ROI segmentation was performed by Dr. A.

Then, one-way analysis of variance (ANOVA) and least absolute shrinkage and selection operator (LASSO) logistic regression were used to select the most useful predictive radiomics features from the training set. For the LASSO algorithm, the optimal penalization coefficient lambda (λ) was set by five-fold cross-validation, and radiomics features with non-zero coefficients within the training set were finally selected to construct the radiomics signature (33–35). The radiomics signature score (Rad-score) formula was generated using a linear combination of the selected features, which were weighted by their respective coefficients. Then, the Rad-score was calculated for each patient using this formula to compare the significant difference between the Rad-score of sub-1 cm benign and malignant lesions in the training and validation sets (Mann–Whitney U test). The predictive efficiency of the radiomics signature was quantified by using the area under the receiver operating characteristic (ROC) curve (AUC) in the training and validation sets.

### Clinical Predictive Factors Selection and Radiomics Nomogram Construction

One-way ANOVA and multivariate logistic regression were performed to select independent predictive factors related to the identification of benign and malignant thyroid lesions, including clinical characteristics and Rad-score in the training set. Then, a radiomics nomogram was constructed on the basis of the multivariate logistic regression model.

### Performance of the Radiomics Nomogram

ROC curves were plotted to assess the discrimination performance of the radiomics nomogram for sub-1 cm benign and malignant lesions. The calibration performance of the radiomics nomogram was evaluated by using calibration (agreement between the observations and the predicted malignant probability) curve. The main and ultimate purpose of using the nomogram is to combine the research results with clinical decisions so as to maximize patient benefit. However, discrimination and calibration could not capture the clinical consequences of a particular level of discrimination or degree of miscalibration. Therefore, decision curve analysis (DCA) was conducted to determine the clinical usefulness of the radiomics nomogram by quantifying the net benefits at different threshold probabilities in the validation set (net benefit is defined as true-positive rate minus false-positive rate, weighted by the relative harm of false-positive and false-negative results).

### Statistical Analysis

All statistical analyses were performed using R software 3.5.3 and Python 2.7 software. Continuous data are reported as mean ± standard deviation or median (interquartile range). Categorical data are reported as numbers (%). All the levels of statistical significance were two-sided, and *P*-values < 0.05 were considered significant. The “SelectKBest” and “LassoCV” in Scikit-learn were used for selecting radiomics features. The “glm” function was used for multivariate logistic regression analysis. The “glmnet” package was used for LASSO logistic regression. The “vioplot” package was used to plot the violin diagram. The “Hmisc” package was used to draw the radiomics nomogram. The “pROC” package was used to plot the ROC curves and measure the AUCs. The “rms” package was used to plot the calibration curves. The “rmda” package was used to perform DCA.

## Results

### Clinical Characteristics

The clinical characteristics of patients in the training and validation sets are summarized in **Table 1**. No significant differences were found between the training and validation sets in terms of gender, TI-RADS category, CT characteristics (maximum diameter, calcification, and location of nodule), age, FT3, FT4, TSH, or pathology (*P* > 0.05).

**Table 1:** Clinical characteristics of patients in the training and validation sets.

	Training set (n=136)	Validation set (n=35)	P-value
**Gender**			0.144
Male	40(29.41)	6(17.14)	
Female	96(70.59)	29(82.86)	
**TI-RADS**			0.138
3	5(3.68)	4(11.43)	
4A	31(22.79)	4(11.43)	
4B	82(60.29)	25(71.43)	
4C	17(12.50)	2(5.71)	
5	1(0.74)	0(0.00)	
**CT-location**			0.708
Left	69(50.74)	19(54.29)	
Right	67(49.26)	16(45.71)	
**CT-calcification**			0.668
Yes	23(16.91)	7(20.00)	
No	113(83.09)	28(80.00)	
**Age(years),mean ± SD**	46.21 ± 11.18	47.46 ± 10.54	0.554
CT-diameter(cm)^*^,mean ± SD	0.64 ± 0.19	0.59 ± 0.19	0.184
**FT3,mean ± SD**	4.95 ± 0.68	5.27 ± 1.77	0.088
**FT4,mean ± SD**	16.19 ± 2.38	16.96 ± 4.52	0.171
**TSH,mean ± SD**	2.41 ± 1.24	2.60 ± 1.85	0.489
**Nodule pathology**			0.828
Benign	44(32.35)	12(34.29)	
Malignant	92(67.65)	23(65.71)	

TI-RADS, Thyroid imaging reporting and data system; CT, computed tomography; SD, standard deviation; FT3, free triiodothyronine; FT4, free thyroxine; TSH, thyroid stimulating hormone; Data are number of patients and percentage if not specified.

^*^Largest diameter of the target lesion.

### Construction of Radiomics Signature

**Figure 4:**
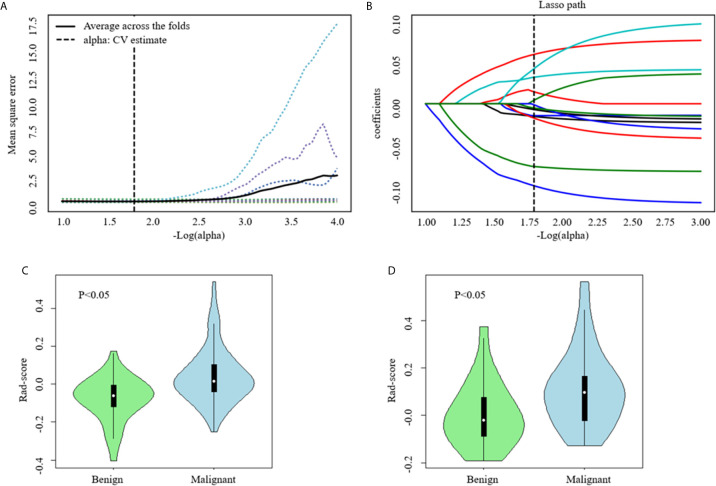
Computed tomography (CT) image features selection using one-way analysis of variance (ANOVA) and the least absolute shrinkage and selection operator (LASSO) logistic regression model in the training set. **(A)** The five-fold cross-validation and the minimal criteria process was used to generate the optimal penalization coefficient lambda (λ) in the LASSO model. The vertical line define the optimal values of λ, where the model provides its best fit to the data. The optimal λ value of 0.165 with -log (λ) =1.8 was selected. **(B)** LASSO coefficient profiles of the radiomics features. The vertical line was drawn at the value selected using five-fold cross-validation, where optimal λ resulted in 13 nonzero coefficients. Violin distribution of Rad-score for benign and malignant nodules in the training **(C)** and Validation **(D)** Sets. Green violin plots show data distribution of benign nodules, blue ones data distribution of malignant nodules. In each violin plot, the white point represents the median value of each group; the vertical black line represents the range.

In the training set, a total of 1409 radiomics features were extracted from each CT image. Favorable inter-observer and intra-observer reproducibility of feature extraction was achieved with intra-observer ICCs ranging from 0.856 to 0.914 and inter-observer ICCs ranging from 0.817 to 0.897. Then, 13 non-zero coefficient features associated with benign and malignant lesions were selected after using the ANOVA and LASSO algorithms (**Figures 4A, B** and **Table 2**), which included one morphological feature, six first-order features, and six texture features. Rad-score of each lesion was calculated by the 13 radiomics features. The results showed that the Rad-scores (median [interquartile range]) of the malignant lesions and benign lesions were significantly different (0.02 [-0.06 to 0.10] *vs.* -0.07 [-0.12 to 0.00], respectively, P < 0.05, Mann–Whitney U test); this difference was confirmed in the validation set (0.07 [-0.03, 0.15] *vs.* 0.03 [-0.08, 0.01], respectively, P < 0.05). The violin distribution of Rad-scores for benign and malignant lesions in the training and validation sets is presented in **Figures 4C, D**.

**Table 2:** Radiomics features selected in ANOVA and LASSO regression analysis.

Radiomics features	Coefficients
original_shape_Sphericity_pv	-0.099838	
original_firstorder_Kurtosis_pv	0.060079	
original_firstorder_Range_pv	0.032309	
logarithm_firstorder_Energy_pv	-0.017484	
original_firstorder_TotalEnergy_pv	-0.015415	
original_firstorder_Energy_pv	-0.014501	
logarithm_firstorder_Skewness_pv	-0.006939	
logarithm_gldm_DependenceNonUniformity_av	-0.076419	
original_glrlm_RunEntropy_pv	0.042593	
original_glszm_SmallAreaEmphasis_pv	0.014181	
original_glrlm_RunLengthNonUniformity_pv	-0.005117	
original_glszm_SmallAreaLowGrayLevelEmphasis_pv	0.004510	
original_gldm_DependenceNonUniformity_pv	-0.001145	

Thirteen radiomics features with non-zero coefficients in one-way analysis of variance (ANOVA) and the least absolute shrinkage and selection operator (LASSO) logistic regression model were selected. The radiomics signature was constructed based on the regression analysis with a radiomics score calculated for each patient. The formula to calculate the score of radiomics signature is Rad-score = Radiomics features× Coefficient.

### Construction of Radiomics Nomogram

**Figure 5:**
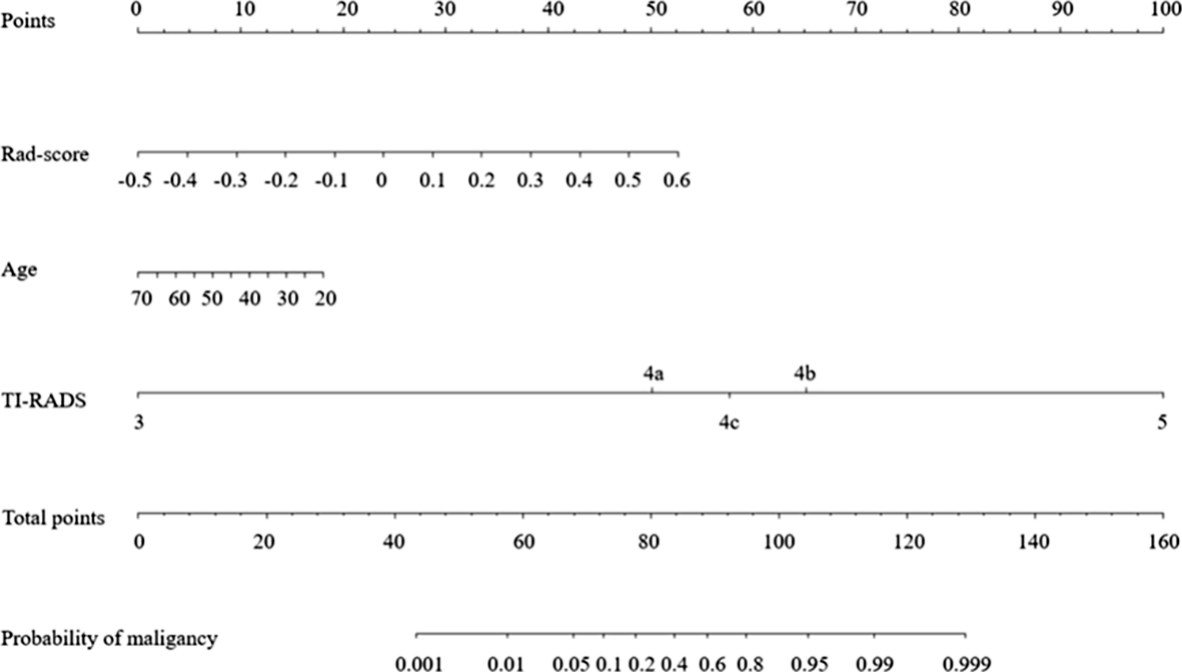
Radiomics nomogram for the prediction of benign and malignant thyroid nodules of sub-1cm.

After performing one-way ANOVA and multivariate logistic regression, age, TI-RADS category, and Rad-score were identified as final predictors of sub-1 cm thyroid malignancy. A radiomics nomogram incorporating these three predictors was constructed (**Figure 5**).

### Performance of Radiomics Nomogram

**Figure 6:**
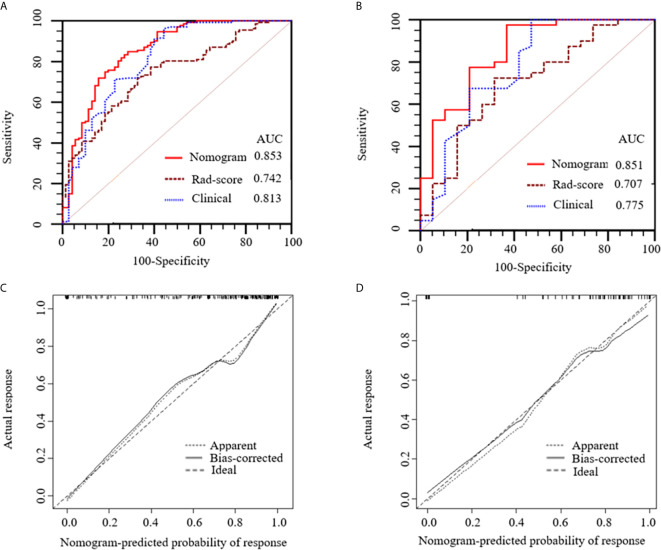
Receiver operating characteristic (ROC) curves of the nomogram (red lines), Rad-score model (brown lines) and clinical model (blue lines) in the training **(A)** and validation **(B)** sets, respectively. Calibration curves of the nomogram in the training **(C)** and validation **(D)** sets, respectively. The diagonal dotted line represents an ideal prediction, while the solid lines represent the performance of the nomogram. Closer fit to the diagonal dotted line indicates a better prediction.

**Figure 7:**
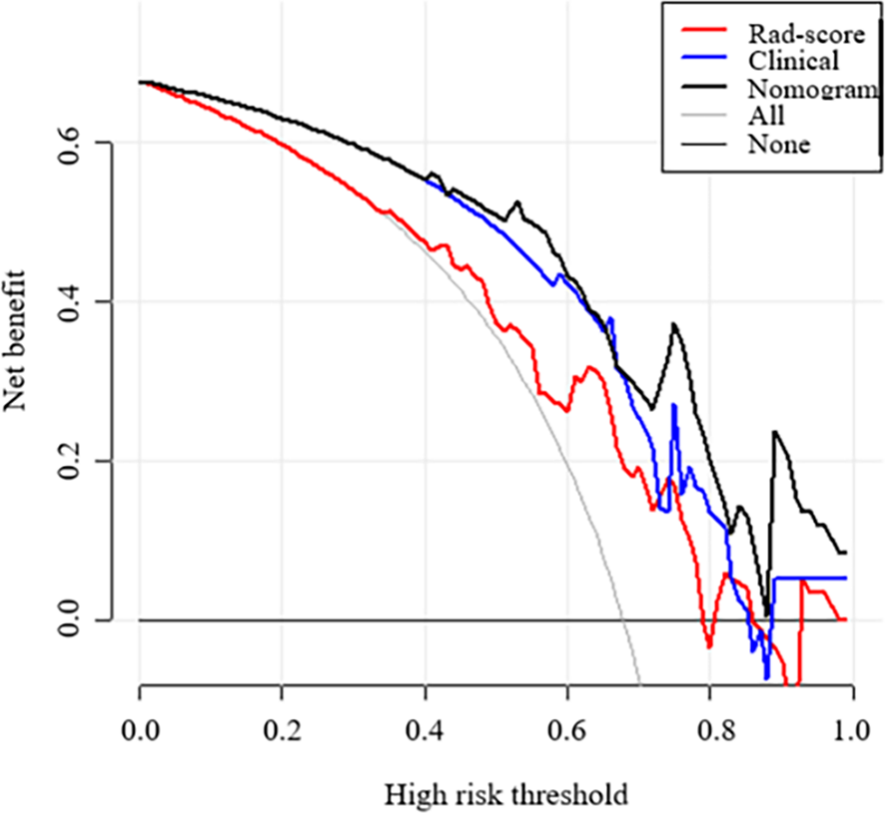
Decision curve analysis (DCA) of each model derived from the validation set. The y-axis measures the net benefit. The x-axis shows the corresponding risk threshold. The grey line represents the assumption that all lesions were malignant. The black line represents the assumption that all nodules were benign. If the threshold probability was more than 40%, using the nomogram to predict malignancy added more benefit than the Rad-score model (red line) and clinical model (blue line).

**Figures 6A, B** show the ROC curves of the nomogram, Rad-score, and clinical prediction model in the training and validation sets. The results of AUCs for the nomogram, Rad-score, and clinical prediction model were 0.853 (95% confidence interval [CI]: 0.797, 0.899), 0.742 (95% CI: 0.676, 0.801), and 0.813 (95% CI: 0.752, 0.864) in the training set and 0.851 (95% CI: 0.735, 0.931), 0.707 (95% CI: 0.574, 0.818), and 0.775 (95% CI: 0.648, 0.873) in the validation set, respectively. The sensitivity and specificity of three models in the training and validation sets were exhibited in the **Table 3**, which showed the radiomics nomogram had good discrimination efficiency. **Figure 6C** illustrates the calibration curve of the radiomics nomogram. The calibration curve showed good calibration in the training set. The favorable calibration of the radiomics nomogram was confirmed with the validation set (**Figure 6D**). DCA was used to assess the clinical usefulness of the radiomics nomogram, radiomics signature, and clinical prediction model in the validation set (**Figure 7**). If the threshold probability of clinical decision was between 0.0 and 1.0,using the nomogram to predict malignancy provided more benefit than either the treat-all (assuming all lesions were malignant) or treat-none strategy (assuming all lesions were benign). Moreover, the use of radiomics nomograms to predict malignancy provided more net benefit than the use of the radiomics signature alone or clinical prediction model alone.

**Table 3:** Predictive performance of three models.

Model	Training set			Validation set		
	AUC (95%CI)	Sensitivity	Specificity	AUC (95%CI)	Sensitivity	Specificity
**Nomogram**	0.853	0.803	0.757	0.851	0.775	0.79
	(0.797-0.899)			(0.735-0.931)		
**Rad-score**	0.742	0.720	0.671	0.707	0.725	0.684
	(0.676-0.801)			(0.574-0.818)		
**Clinical**	0.813	0.735	0.671	0.775	0.675	0.632
	(0.752-0.864)			(0.648-0.873)		

## Discussion

In recent years, the morbidity of PTMC has dramatically increased. Studies have shown that some PTMCs can be associated with highly aggressive histological variants and even exhibit early localized invasion or lymph node and distant metastasis (36–39). Unfortunately, the accuracy of diagnosing PTMC is inefficient, resulting in a proportion of patients being mistreated or misdiagnosed. In the present study, the potential ability of CT-based radiomics for identifying sub-1 cm benign and malignant thyroid lesions was discussed. Our results indicated that the radiomics nomogram combined with radiomics signature and clinical risk factors could preoperatively predict small thyroid lesions with good performance.

To construct the radiomics signature, a LASSO logistic regression model was used to reduce the radiomics features. This method is widely used in discriminating benign and malignant lesions (37, 40, 41), and it is designed to avoid overfitting (42, 43). In our study, 13 radiomics features were finally selected as the most closely related features to the sub-1 cm thyroid lesion status, including 1 shape feature, 6 first order statistics features, 2 gray level dependence matrix (GLDM)-derived texture features, 2 gray level run-length matrix (GLRLM)-derived texture features, and 2 gray level size zone matrix (GLSZM)-derived texture features. Among them, sphericity accounted for the greatest weighted, indicating that the shape feature of the lesion may be the most important feature affecting the diagnosis of sub-1 cm benign and malignant thyroid lesions. Several studies have shown that shape features differentiate benign and malignant lesions on the basis of CT scans (44–46). At the same time, sphericity was inversely correlated with the radiomics signature, which is consistent with the findings of Limkin et al. (47). The radiomics signature based on unenhanced and arterial contrast-enhanced CT images showed good discrimination ability in the training (AUC: 0.742) and validation sets (AUC: 0.707).

It is well known that US and US-FNAB have significant advantages in determining thyroid lesions, but Zhang et al. (48) found that the sensitivity, specificity, and AUC of US in identifying PTMC were only 0.684, 0.771 and 0.728, which means that the diagnostic ability of US in PTMC is varies greatly. On the other hand, US-FNAB did show a greater advantage in the diagnosis of PTMC, Gao et al. (49) showed that the sensitivity, specificity, and AUC of US-FNAB in identifying PTMC were 0.988, 0.905, and 0.947, respectively. As a contrast, in our study, the sensitivity, specificity, and AUC of the nomogram model were 0.775, 0.790, and 0.851, respectively. However, CT is a more objective and non-invasive option.

The present study indicated that age and TI-RADS category were significant predictive factors. However, whether age is one of the important clinical risk factors of PTC still remains to be elucidated. Chen et al. (50) recently provided evidence that age, margin, shape, echogenic foci, echogenicity, and nodule halo sign are independent risk factors, whereas Liang et al. (51) reported that age has no significant relevance with PTC diagnosis.

The present study has several strengths. First, an independent validation set was used to verify the discrimination ability of the nomogram model. The results also had good diagnostic ability (AUC: 0.851), which demonstrates that the nomogram model has good generalization ability. However, the previous study failed to determine the usefulness of the radiomics nomogram in the clinical setting (52, 53). Hence, DCA was used to assess whether the radiomics nomogram could improve individual benefit. The results showed that if the threshold probability was more than 0, the predictive ability of the radiomics nomogram was more favorable than that with or without patient treatment. To improve the feature recognition rate, the gray value of ROI was standardized, discretized, and resampled. It effectively improved the repeatability of the research results. Finally, to comprehensively reflect the radiomics features of PTMC, unenhanced and arterial contrast-enhanced CT images were extracted for radiomics analysis.

This study has several limitations. First, bias is inevitable as the present study is retrospective in nature. Prospective studies are needed to control for confounding variables. Second, this study utilized a single center and had a small sample size. A large sample size and multiple centers are needed to improve the efficiency of the model. Third, a manual method is applied to image segmentation. Although manual segmentation is the gold standard, it may increase the variability of feature extraction. To avoid this disadvantage, the consistency of feature extraction was validated by using ICC.

In conclusion, this study presents a noninvasive predictive tool that incorporates CT radiomics signature and clinical risk factors. The radiomics nomogram shows favorable predictive accuracy in identifying sub-1 cm benign and malignant thyroid lesions. Multicenter retrospective validation and prospective randomized clinical trials should be performed in subsequent studies to obtain high-level evidence for the clinical application of this nomogram.

## Data Availability Statement

The original contributions presented in the study are included in the article/supplementary material. Further inquiries can be directed to the corresponding authors.

## Ethics Statement

This study was approved by the ethics committee of the Yantai Yuhuangding Hospital.

## Author Contributions 

XW and JL implemented the literature searching, and manuscript writing. YM implemented data collecting, ROI segmentation literature searching, and manuscript writing. YY contributed to data analysis and figures making. JC implemented the algorithm and software development. NM identified the radiological characteristics of PTMC, and estimated and adjusted the accuracy of ROIs. XS conducted the design, quality control, and data interpretation of this study. All authors contributed to the article and approved the submitted version.

## Funding

This study was supported by the “Taishan Scholar” Project (No. ts20190991).

## Conflict of Interest

JC was employed by Huiying Medical Technology Co., Ltd.

The remaining authors declare that the research was conducted in the absence of any commercial or financial relationships that could be construed as a potential conflict of interest.
